# Involving relatives in relapse prevention for bipolar disorder: a multi-perspective qualitative study of value and barriers

**DOI:** 10.1186/1471-244X-11-172

**Published:** 2011-11-01

**Authors:** Sarah Peters, Eleanor Pontin, Fiona Lobban, Richard Morriss

**Affiliations:** 1 School of Psychological Sciences, University of Manchester, UK; 2 School of Population, Community and Behavioural Sciences, University of Liverpool, UK; 3 Spectrum Centre for Mental Health Research, University of Lancaster, UK; 4 Division of Psychiatry, University of Nottingham, UK

## Abstract

**Background:**

Managing early warning signs is an effective approach to preventing relapse in bipolar disorder. Involving relatives in relapse prevention has been shown to maximize the effectiveness of this approach. However, family-focused intervention research has typically used expert therapists, who are rarely available within routine clinical services. It remains unknown what issues exist when involving relatives in relapse prevention planning delivered by community mental health case managers. This study explored the value and barriers of involving relatives in relapse prevention from the perspectives of service users, relatives and care-coordinators.

**Methods:**

Qualitative interview study nested within a randomized controlled trial of relapse prevention for individuals with bipolar disorder. The purposive sample of 52 participants comprised service users (n = 21), care coordinators (n = 21) and relatives (n = 10). Data were analyzed using a grounded theory approach.

**Results:**

All parties identified benefits of involving relatives in relapse prevention: improved understanding of bipolar disorder; relatives gaining a role in illness management; and improved relationships between each party. Nevertheless, relatives were often discouraged from becoming involved. Some staff perceived involving relatives increased the complexity of their own role and workload, and some service users valued the exclusivity of their relationship with their care-coordinator and prioritized taking individual responsibility for their illness over the benefits of involving their relatives. Barriers were heightened when family relationships were poor.

**Conclusions:**

Whilst involving relatives in relapse prevention has perceived value, it can increase the complexity of managing bipolar disorder for each party. In order to fully realize the benefits of involving relatives in relapse prevention, additional training and support for community care coordinators is needed.

**Trial registration:**

ISRCTN41352631

## Background

Clinical guidelines recommend structured psychological interventions should be offered as an adjunctive intervention to psychopharmacology to prevent relapse for bipolar disorder [1]. Relapse prevention (RP) teaches individuals to recognize and manage the early warning signs and triggers to their mania and depressive episodes. In doing so individuals are forewarned of the recurrence of a relapse in time to seek early treatment and so minimize serious harm [2]. This approach is effective in improving function, increasing time to relapse and reducing the percentage of people hospitalized: recommendations are that mental health services should routinely provide RP to adults with bipolar disorder [3].

The role of relatives in RP is less clear. Relatives of people with bipolar disorder experience high levels of burden which are associated with physical and mental health problems and increased use of medical and mental health services [4], particularly amongst caregivers living with patients [5]. Among people with bipolar disorder, there is a perception that carers and families are often excluded from management decisions and ignored by health professionals to the distress of family members who remain uninformed about bipolar disorder [6]. Most families report wishing for support and education from services, but that they rarely receive it [7]. Under these circumstances, families cannot be expected to be as effective as they might be in detecting clinical signs of illness and obtaining help. There are several mechanisms through which relatives' involvement can support service users. Relatives can impact positively on the outcome for patients by providing structures that encourage stable routines and emotional self-regulation strategies [3]. Conversely, relatives' expressed emotion is a robust predictor of relapse in psychiatric conditions, particularly mood disorders [8]. High expressed emotion has been associated with dysfunctional patterns of communication [9] and blaming attributions for negative patient-related events [10].

Together these findings have prompted a growing field of research into interventions at the level of the family to reduce carer burden, develop more helpful illness attributions and patterns of family communication, improve medication concordance and reduce relapse rates. A systematic review of interventions involving relatives was unable to draw conclusions due the heterogeneity and limited size of trials [11]. Nevertheless, recent trials conducted in families of adults [12] and adolescents [13] or carers alone [14] have yielded positive effects on outcome, illustrating the potential value of involving relatives to improve the outcome of bipolar disorder. It has been recommended that engaging families in helping patients to recognize individual early warning signs of mania or depression is a helpful adjunct to pharmacological management [1,3].

Typically, however, research into relapse prevention interventions for bipolar disorder has not specifically sought to involve relatives or carers, but has assessed individualized treatment delivered through specialist services, expert therapists or extensive therapy [15-17] none of which are routinely available in the mainstream services such as the UK National Health Service (NHS). Moreover, RP planning is most useful when patients are well, which is a time when they are likely to have limited contact with medical or mental health specialists. During these periods, service users' primary contact will be a designated member of their community mental health team, who is responsible for their case management. These care coordinators are typically from a nursing, occupational therapy or social work background and will have limited opportunities for specialist training in specific psychological interventions for bipolar disorder [18]. This model is typical within the UK NHS for community follow-up care for people with serious mental illness, and is increasingly found in many over services across the world [19].

A key advantage of RP is that, compared to more sophisticated approaches involving early warning signs (such as some forms of cognitive behaviour therapy and family therapy) simple RP interventions can be taught more quickly and easily to both non-specialist health professionals without requiring extensive training in psychotherapy [20]. A recent trial found that RP could be taught to care coordinators and that this improved social functioning compared with treatment as usual amongst service users with bipolar disorder [21].

Consequently opportunities to involve relatives in relapse prevention planning are likely to most usefully involve care coordinators, who are not trained in family therapy and may not recognize the potential benefit of engaging family members in patients' care planning. Attempts to involve relatives in relapse planning have however been met with limited success [21]. If the potential benefits of involving relatives in RP is to be achieved within routine care, it is important to understand the value health professionals, patients and relatives see (if any) in involving family members in relapse prevention planning, and what barriers exist that deter relatives from taking a greater role.

This paper reports the findings of a qualitative study examining the views of service users, relatives and care-coordinators of the value and barriers of involving family members in relapse prevention.

## Methods

### Study context

The study employed a qualitative approach that was nested within a cluster randomized controlled trial that had provided an opportunity for care coordinators to involve relatives in relapse prevention planning for service users with bipolar disorders. The aim of the trial was to assess the feasibility of training care coordinators (CCs) to offer a relapse prevention (RP) to individuals with bipolar disorder and, where possible, a relative [21,22]. The trial provided an ideal context within which to examine the views of relatives, care coordinators and service users about their experiences of involving relatives in relapse prevention planning, and to ascertain the potential benefits and barriers to developing and implementing this role within routine clinical practice [23]. During the trial 112 CCs from 23 Community Mental Health Teams (CMHTs) in the North West of England, UK were recruited and referred 96 service users (SUs). Full details of recruitment to the trial are reported elsewhere [21]. CCs were randomly allocated by CMHTs to receive training in RP (n = 56) or to continue to offer treatment as usual (TAU, n = 40). Intervention was delivered by CCs to SUs and their relative. Relatives were eligible to take part in the trial if they were aged 18 or above and had a minimum of two face-to-face weekly contacts totaling ≥ 10 hours. Service users were given the option of inviting a relative to take part if they wished, but they were not required to do so. Ethical approval was obtained through the Central Office for Research Ethics Committees (COREC).

**Figure 1:**
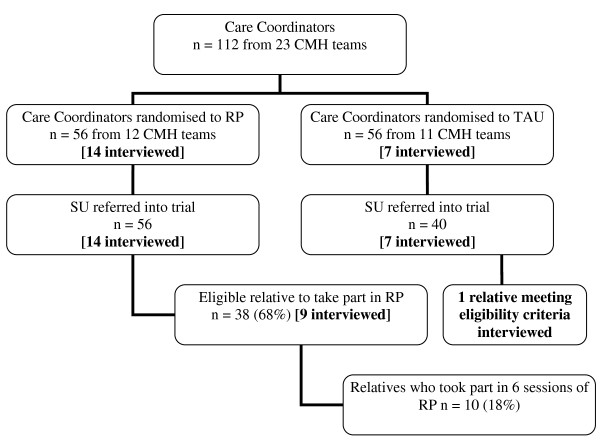
**Flow diagram of trial participants**. Sample interviewed for qualitative study in parentheses.

Of the 56 SUs who were trained in ERP, 38 (68%) had a relative who was eligible to take part in the intervention. Of these, 10 (26% of eligible relatives) relatives fully took part in all six sessions of the relapse prevention intervention (See Figure 1).

### Sampling

CCs, SUs and relatives involved in the trial formed the strategic sampling pool for this qualitative study [21]. Purposive sampling was used to select participants for interviews to ensure a full range of views were represented. CCs were selected to ensure a range of experience of training clients in RP and different occupational backgrounds. SUs were selected to ensure across participants were represented on key variables: whether or not they had a relative involved in training, whether or not they had a relapse since baseline and time since diagnosis. All relatives included met eligibility criteria for the trial. Nine had a relative allocated to RP and five of these had chosen to take part, one was unsure and three had declined the opportunity. A further relative was recruited from the TAU group and so had not had an opportunity to be involved. All those approached agreed to be interviewed. The final sample comprised 21 care coordinators, 21 service users and 10 relatives (See Tables 1, 2 and 3 for participant details).

**Table 1:** Summary clinical and demographic information of care coordinators interviewed (n = 21)

Characteristic	n (%)
*Group*	
relapse prevention	14 (67)
treatment as Usual	7 (33)

*Sex*	
female	14 (67)
male	7 (33)

*Age(years)*	mean 45 (range 29-57)

*Professional background*	
community psychiatric nurse	18 (86)
occupational therapist	2 (10)
social worker	1 (4)

Years worked in community mental health team	mean 7.2 (range 1-30)

Deprivation indices of work place*	

lower quartile (least deprived)	4 (19)
mid lower quartile	5 (24)
mid upper quartile	1 (5)
upper quartile (most deprived)	11 (52)

*Caseload balance*	

number of SUs with bipolar diagnosis	mean 6 (range 1-9)
% of caseload with bipolar diagnosis	mean 20% (range 3-40%)
number of SU receiving intervention**	mean 2 (range 0-3)

* Postcodes were converted into Townsend deprivation indices [32] and categorised into bands in accordance with deprivation indices for England.

**At time of interview. RP group only

**Table 2:** Summary clinical and demographic information of service users interviewed (n = 21)

Characteristic	n (%)
*Group*	
relapse prevention	14 (67)
treatment as usual	7 (33)

*Sex*	
female	13 (62)
male	8 (38)

*Age (years)*	mean 47 (range 24-63)

Deprivation indices of work place**	
lower quartile (least deprived)	3 (14)
mid lower quartile	4 (19)
mid upper quartile	6 (29)
upper quartile (most deprived)	8 (38)

*Employment status*	
unemployed	10 (47)
part or full-time employed	7 (33)
retired student	2 (10) 2 (10)

*No. of previous episodes*	
Depression (n = 21)	
0-2	5 (24)
3-5	0 (0)
6-10	1 (5)
11-20	4 (19)
>20	5 (23)
unknown	6 (29)
Mania (n = 21)	
0-2	3 (14)
3-5	4 (19)
6-10	2 (11)
11-20	3 (14)
>20	4 (19)
unknown	5 (23)

*Years since first episode*	mean 21 (range 1-46)

Relapsed since RP intervention*	
Relapse Prevention	
Yes	6 (43)
No	8 (57)
Treatment as Usual	
Yes	2 (29)
No	5 (71)

*At time of interview

**Postcodes were converted into Townsend deprivation indices [24] and categorized into bands in accordance with deprivation indices for England.

**Table 3:** Summary information of relatives interviewed (n = 10)

Characteristic	n (%)
*Group*	
relapse prevention	9 (90)
treatment as usual	1 (10)

*Sex*	
female	6 (60)
male	4 (40)

*Relationship to service user*	
spouse	6 (60)
parent	3 (30)
sibling	1 (10)

*Living with service user*	
yes	8 (80)
no	2 (20)

Participation in RP*	
yes	5 (56)
no	3 (44)
unsure	1 (11)

* RP group only (n = 9)

### Procedure

Participants were interviewed by a researcher (EP). SUs and relatives were interviewed in their own homes and CCs in their place of work. Interviews with SUs averaged 60 minutes (range 15-120), CCs 45 minutes (range 25-96) and relatives 48 minutes (range 11-74). All participants gave written informed consent and a topic guide provided a flexible interview framework. SUs and relatives were asked to talk about their experiences of bipolar disorder and taking part in the RP intervention and of the services they received from their mental health team. CCs were also asked to recount their perceived role with SU and relatives, experiences of delivering RP and any issues around involving relatives in RP. Questioning was structured by the interviewer to cover main topics, but was also responsive to issues emerging from participants' accounts. The interviewer used a combination of open questions to elicit free responses, and focused questions for probing and prompting. Emerging themes were explored throughout the data collection process and specifically attended to and developed in further interviews. All interviews were digitally recorded and transcribed verbatim.

### Analysis

A grounded theorizing [24] approach was used to develop conceptual categories from the data. Themes, categories and memos were coded into a word document which was refined and elaborated in light of incoming data and analysis employing an inductive stance. The interviewer conducted the analysis on all interviews. In addition, each interview was separately analyzed by at least one other researcher to check for reliability of coding. Findings and themes were discussed regularly by an interdisciplinary team comprising of researchers with different professional backgrounds (psychological, psychiatric, sociological and nursing) thereby increasing the trustworthiness of the analysis [25]. Analysis and data generation took place in parallel whereby further interviews were sought to test emerging patterns which were modified using constant comparison, 'cycling' between sets of data, the developing analysis and further sampling and interviews. Data generation continued until thematic saturation was achieved. In reporting the final analysis the data are presented to illustrate the range and commonality of meaning of each category.

## Results

The analysis focuses on participants' responses of i) the value placed on involving relatives in RP and ii) the potential barriers to involving relatives routinely in such interventions.

### Value of involving relatives

Participants from each group recognized some value in involving a relative in RP, with benefits identified for relatives, SUs and CCS (See Table 4). Values were 'understanding bipolar disorder', 'relative's role in the management of BD', and 'the relationship between CC, SU and relative (R)'.

**Table 4:** Value and barriers of involving a relative in relapse prevention for relatives, service users and care coordinators

	*For relatives*	*For Service Users*	*For care coordinators*
**Value**	• Increases understanding of bipolar disorder, triggers and EWS • Gives relatives a role - empowering • Recognize need to seek help earlier - important in a crisis • Improves relationship with service users • Improves relationship with mental health services	• Provide insight into triggers and EWS • Another 'pair of eyes' to recognize EWS and triggers • Increased support during a crisis • Improves relationship with relative	• Provide insight into triggers and EWS • Another 'pair of eyes' to monitor service user • Improves contact during a crisis • Improves relationship with relative

**Barriers**	• Conflict with work and other commitments • No suitable relative to take part • Not wanting to intrude on relationship between Service User and Care coordinator	• Want to keep illness and issues private from family • Want to keep family issues private from CC • Did not want to burden family members • Fear increased monitoring will lead to increase misattribution of normal emotions and behaviors • Concern over placing relatives in position of power • Families were a source of stress and trigger to relapse • Relationship with CC is exclusive from relatives	• Takes longer • Informal increase of caseload • Have to maintain confidentiality of service user • Difficulty dealing with family dynamic within sessions • Unconfident as therapists

#### Understanding bipolar disorder

RP was perceived to have increased relatives' understanding of bipolar disorder. Consequently, they were able to make sense of past behavior;

With bipolar...sometimes it's easier with the patients. It's the relatives who can't get their heads round what was going on and why this person suddenly hated them...did all these bizarre things (76: CC)

Through RP relatives gained further understanding of triggers and early warning signs to relapse, distinguishing between emotions and behaviors that were normal and those that were symptoms and required action. This gave them a perception of having some control over events and was experienced as empowering;

For the first time ever I thought, we are not being disempowered, we are being empowered and for families with bipolar. That's rare (63: R)

Both partners and the participants, I think they felt to a degree a little bit more empowered in the situation. That they have a little more knowledge and a little bit more control knowing what may happen. (39: CC)

#### Role in management of bipolar disorder

RP provided relatives with a new role (or legitimized a role they had already been undertaking) - that of monitoring SUs mood and behavior. This was especially valued by CCs who identified relatives as being ideally positioned to act as '*another pair of eyes and ears, doing some monitoring*' (16: CC). SUs also valued relatives being able to help them monitor their mood and behavior. Relatives helped recognize triggers and early warning signs, often contributing information that SUs were unaware of themselves;

My memory is pretty patchy over the things that have happened...she is able to answer questions that I honestly, I can't answer (33: SU)

I would think that fundamentally if you are not really getting somebody else involved in it, you are maybe knocking 30% off the value of what you are doing away because you are losing somebody else having insight into what is happening if these people aren't picking it up even, you know (39: CC)

Inviting relatives to monitor symptoms was perceived as greatly increasing the likelihood of recognizing early warning signs. By being aware of, and recognizing signs to relapse earlier, relatives had an active role in RP. Furthermore, RP helped relatives see the benefits of seeking help from the services at the appropriate time. Consequently they reported feeling less anxious about the prospect of a relapse and hopeful that they were now equipped to be able to recognize early warning signs, intervene and prevent an episode;

I am a lot more confident that I can deal with it if it happened again, and the first thing I would do is make sure that she got a much earlier assessment from the GP and the consultant, and if necessary get her voluntarily into hospital early....I have recognized now that the longer it goes on the worse it goes. (35: R)

#### Relationships between CC, SU and relative

All groups felt that relationships between family members, CCs and services were pivotal. Taking part in an RP intervention provided relatives with an opportunity to be much more involved in service provision and forge a relationship with the CC;

*I have more contact with the family since *[doing RP]... [Previously] *the partners just tended to busy themselves in the kitchen (39: CC)*

CCs reported relatives being much more actively involved since the intervention. By having an opportunity to engage with and develop a rapport with services, relatives knew who to contact within the care team and had confidence that they would be listened to. This is especially important during a crisis as relatives were often the first point of contact with services;

Now I feel very much part of it as well, and I know that I can ring up...If he was becoming ill I would ring up and ask the CPN to come round. She is very good (33: R)

As well as the potential to enhance the relationship between family members and CCs, RP could also improve relationships between SUs and relatives. RP provided an opportunity to talk together about past events, and the impact of bipolar disorder on themselves and the family;

*I think he *[service user] *was quite relieved really that he could discuss things with her *[wife] *that he hadn't talked about in the past. And quite relieved that she had a better understanding of how he was (28: CC)*

Some CCs reported that an increased understanding between the SU and their relative had led to their relationship being less stressful and pressured and that as a consequence novel information was shared;

It's very good...to get the carer and the client actually sitting together and talking about it...you know quite a few things that came out of it. (14: CC)

However, this was not always that case; sometimes information would be withheld *because *relatives were present;

I suppose some of the things that he may have discussed if we had been in our normal therapeutic sessions. We didn't discuss some things. (28: CC)

### Barriers to involving a relative in relapse prevention

Despite the various benefits RP could bring to the individuals, a range of barriers to involving a relative in RP were identified by all groups of participants.

#### Time

A reason often cited by relatives for why they hadn't chosen to get involved in RP was lack of time or work commitments. CCs often visit SUs during the working day, making it practically difficult for some family members to attend sessions. However the same relatives also described elsewhere how RP could *save *time by preventing a relapse;

If you nip it in the bud, it can save you months and months of heartache (31: R)

The data revealed far more complex reasons for relatives not being involved in RP. These were associated with; the family dynamic; autonomy and privacy; and professional burden.

#### Family dynamic

For some SUs a suitable family member could not be identified to take part in RP. Thirty-two per cent of the trial sample did not have a relative who they had sufficient face-to-face contact with to be eligible for the intervention. Others had relatives with whom they had the required 10 hours contact per week, but felt giving their relative the role of 'carer' was inappropriate;

*My younger sister didn't *[take part]...*it's difficult with her because she is sort of quite looked-up to me...she is 14 years younger than me, so she is more my little sister and I am her big brother type of thing. (20: SU)*

Families could also themselves be a source of stress and a trigger to relapse. Many described hostile and critical relatives who they distrusted to take on decision-making responsibilities;

On all of the occasions that I have been sectioned they have asked me who do you want as your loco parenti, next of kin? And I have always said 'duty social worker'. I would never, ever, ever have my mother and father involved in decisions regarding my care (19: SU)

Involving critical families also raised concerns as it placed them in a position of power which they might manipulate;

*There is odd times jokingly she *[mother] *will say to me I am going to ring *[care coordinator]. *I know what that means, so I go quiet (13: SU)*

#### Autonomy and privacy

Some SUs described wanting to keep their illness, and their management of their illness, private from their family. For some this was because of the stigma of the condition;

I have never talked to them about my symptoms or anything like that...they probably wouldn't understand...maybe they would think I was a freak or something (9: SU)

Others did not want to burden their relative with decisions (*I will carry that load myself; 21: SU) *or problems and felt they alone were responsible for managing their illness. Some were fearful that by increasing a relatives' role in monitoring symptoms they could potentially become overly watchful and misattribute normal emotions as early warning signs which could exacerbate matters.

Her family take a keen interest in her sleep and her mood and sometimes you can see that she gets quite frustrated with that. I mean one bad night and they magnify it and she kind of wants to minimise them but they maximise them. (33: CC)

Many SUs were unused to having their family member involved in their relationship or in contact with mental health services. Generally, the relationship with CCs was valued by SUs and each party recognized that at times it would indeed be inappropriate to involve relatives;

You do need time alone with the client because some of the pressure in their life might actually be with their partner, or there might be things that are happening in their life, they don't want their partner to know about, so you do need that time with them themselves (39: CC)

*I wanted to talk to her *[CC] *the other week but *[husband] *was sitting there... I didn't talk and then as she was leaving I just said I needed to talk to you today, she said ok, well you are coming down Thursday anyway aren't you she said, we will have a talk then (35: SU)*

Relatives also described feeling uncomfortable about 'intruding' on the established relationship between SU and CCs. Additionally, there were family issues that SUs were reluctant for their relatives to discuss with their CC, particularly since they had a less well-established relationship;

*They *[parents] *did have the option to do it, but I wouldn't let them...when I were 14 there was something what happened in my family...I don't think it were fair...for my mom to talk about it (14: SU)*

Ultimately for CCs, if SUs didn't want their relative involved, they had to respect that and abide by their wishes.

*Some people don't want any confidential details discussed with the family...you can't do it without their permission *(*6: CC)*

#### Professional burden

Having a relative present during sessions and the difficulty in maintaining SUs confidentiality was something CCs needed to manage;

*It's very personal...there was a lot of drugs involved I think that her mom didn't know about, and I did explain that we could actually omit that part...she didn't have to know about that part. But...she still didn't want to do it *[involve mother in RP]. *(48: CC)*

For many CCs, delivering RP with a patient was already a new role and added burden to their workload and time. Involving relatives involved further additional work. Firstly it was perceived to increase the frequency and lengths of visits to their client;

You are explaining each session, that takes up a lot and part of it we did with [SU's] husband involved because he was critical anyway of her illness, that took longer because then he wanted explanations. It actually did him good because it changed his attitude about the illness...but it did take a long time (76: CC)

Secondly it was viewed as informally increasing their caseload since in effect they were taking on relatives as clients;

*She *[relative] *was quite tearful and 1 or 2 of the sessions I saw her after the session as well for some individual support. So it increased my work load *(*28: CC)*

Sessions themselves became more complex when family members were present. At times it was difficult to keep the focus on RP as relatives often wanted to talk about their own problems and needs. In addition, managing family dynamics such as guilt, shame and disagreements was reported as being challenging for many CCs;

They argued in the session, so that was difficult managing that, there was a lot of blame within that session (28: CC)

As a consequence CCs sometimes did not encourage their involvement;

So when the person sort of says no I don't want family involved...I think you would be very tempted to sort of brush over it and say, great ok, no problem, because you think God how am I going to cope with them anyway (48: CC)

On the whole CCs were very experienced, with an average of seven years working in the community mental health team and having an average of six clients with BD on their caseload (See Table 2). However, the role of *therapist *was evidently novel and many were unconfident in working with families in this way. Consequently there was a level of concern about being observed performing in this role;

*I think it could be that care coordinators don't encourage it *[relative's involvement] *enough, they feel unsure of it themselves, or standing there doing it on your own, I don't know whether that *[involving a relative] *would be just a bit too much *(*48: CC)*

This was something that relatives were also aware of. In this example a relative, who felt discouraged to take part in the intervention, reflected that it may have been because the CC felt threatened by the relative's own experience as a more experienced therapist.

My feeling was, she didn't really want me to be there...because of my background in counseling (63: R)

## Discussion

This is the first study to examine from a multi-perspective the perceived issues around involving relatives in relapse prevention for bipolar disorder. Clear benefits were described by service users, care coordinators and relatives. In particular in terms of improving communication between relatives and SUs, and between relatives and services, increasing each party's understanding of the disorder and engaging relatives in the role of monitoring symptoms. These are issues that relatives of individuals with bipolar disorder struggle with and currently feel health professionals fail to provide support for [26]. However, several barriers were discovered that might prevent successful involvement of relatives in relapse prevention: the family dynamic, the autonomy and privacy of the SU and the professional burden of the health professional. These barriers increased the complexity of involving relatives in the delivery of RP for CCs, SUs and their relatives and they need to be addressed in order for the benefits of relative involvement in psychological interventions to be part of routine care.

The quality of the relationships between relatives and service users had the potential to simplify or increase the complexity of delivering relapse prevention. It is known that families of individuals with bipolar disorder often experience high levels of family burden and criticism, similar to families of individuals with other long term mental health problems [5] and often feel isolated in attempting to balance their own needs with those of their family members' and unsupported by professionals [27]. Our findings suggest even with further trial evidence of the effectiveness of involving families in relapse prevention, this may not be appropriate (or possible) until the needs of carers and relatives are also addressed. Moreover, that health care professionals should receive appropriate training and support to provide this. To engage family members, health professionals may firstly need to establish independent relationships with family members before attempting to engage them in systemic interventions. Relatives may first require individual support to address their own mental health needs. Interventions may also be needed that aim to improve communication and day to day interactions within the family. A clear rationale for family involvement and the specific role of the relative is also crucial. Research has demonstrated that whilst family members desire more involvement and education [7] they can feel that health professionals' focus on encouraging service user autonomy renders them powerless and without a role [28]. Our study demonstrates that preserving autonomy and privacy is bidirectional and similarly important for service users and relatives and is another potential barrier to involving relatives in relapse prevention planning. These tasks may be complex and require therapists with skills, time and appropriate supervision. In our study, care coordinators were able to see the value of involving relatives in terms of saving input from services in the longer term; however they were concerned about the feasibility of increasing the frequency and duration of visits, and the need to take on relatives as an invisible caseload. Without the support of managers, involving relatives in care in this way is unlikely to be implemented into practice. Further research is needed as to the value placed on relapse prevention (and potential barriers) at the level of the wider team organization [29].

A qualitative approach was employed which provided rich data grounded in stakeholders views and ideas [30]. With 52 participants, the sample was large and thematic saturation was assured. A particular strength of the investigation was the use of triangulation which is a recognized procedure for increasing trustworthiness of qualitative analysis [25]. Triangulation was sought in two ways. Firstly, the study question was approached from the perspectives of service providers, users and relatives. Secondly, the thematic framework was developed and tested by a multidisciplinary team of researchers [30].

Participants were selected from a sample recruited to a trial. This was opportunistic as it allowed the identification and interviewing of relatives who met criteria for involvement in relapse prevention and from this sample, participants were selected who had taken part as well as those who had the opportunity but had chosen not to. Given the typically poor retention rates in family interventions e.g. [31], this is an important group to access. However, the limitation of recruiting from a trial population is that only service users (and their relatives) who had been referred to the trial could be contacted. It is possible that the participants referred by CCs differed from those not referred; these differences may have revealed further barriers to involving relatives in care. Our findings suggested that the quality of the relationship was important to successful involvement of relatives and that not all relatives were deemed suitable. The small number of relatives who were available to take part in this study meant the analysis could not be extended to make comparisons between individuals within different types of relationships. It is possible that the perceived value and barriers of involving a relative in relapse prevention therapy may differ for spouses compared to parents or siblings. Further research is needed to investigate this further.

## Conclusions

Although there are clinical benefits of involving families in the care of people with bipolar disorder, there is also a need to ensure sufficient training, supervision and resources if these benefits are going to be fully realized. Interventions targeted at involving family members should ensure that the rationale is understood clearly by all parties and that their role is clarified. Whilst the benefits are potentially substantial, it must be recognized that involving families can provide an additional burden to clinicians' caseload and require additional clinical skills. Additional training and support is necessary for health professionals but these costs may be offset by reducing burden on families and facilitating relapse prevention. Further research is needed to establish the clinical and cost effectiveness of involving relatives in relapse prevention approaches and identify the training and supervision requirements for routine care staff to achieve this.

## Competing interests

The authors declare that they have no competing interests.

## Authors' contributions

SP designed the study, led the analysis and wrote the paper. EP conducted the interviews and the initial analysis and contributed to the drafting of the manuscript. FL was principle investigator for the trial within which the study is nested. FL and RM contributed to the analysis and redrafting of the manuscript. All authors contributed to and have approved the final manuscript.

## Pre-publication history

The pre-publication history for this paper can be accessed here:

http://www.biomedcentral.com/1471-244X/11/172/prepub
